# Supplementary material for: BCKDK Inhibitors Directly Antagonize Angiotensin II Type 1 Receptor and Induce Vasodilation Independently of BCAA Catabolism or BCKDK
Source: bioRxiv. 2026 Oct 11:2026.10.06.757051. Preprint. [Version 1] doi: 10.64898/2026.10.06.757051 (PMC13692194; doi:10.64898/2026.10.06.757051)
Supplement: Supplementary file 1 [file NIHPP2026.10.06.757051v1-supplement-1.pdf]

bioRxiv preprint doi: https://doi.org/10.64898/2026.10.06.757051; this version posted October 11, 2026. The copyright holder for this preprint (which was not certified by peer review) is the author/funder, who has granted bioRxiv a license to display the preprint in perpetuity. It is made available under a CC-BY 4.0 International license.

Figure S1

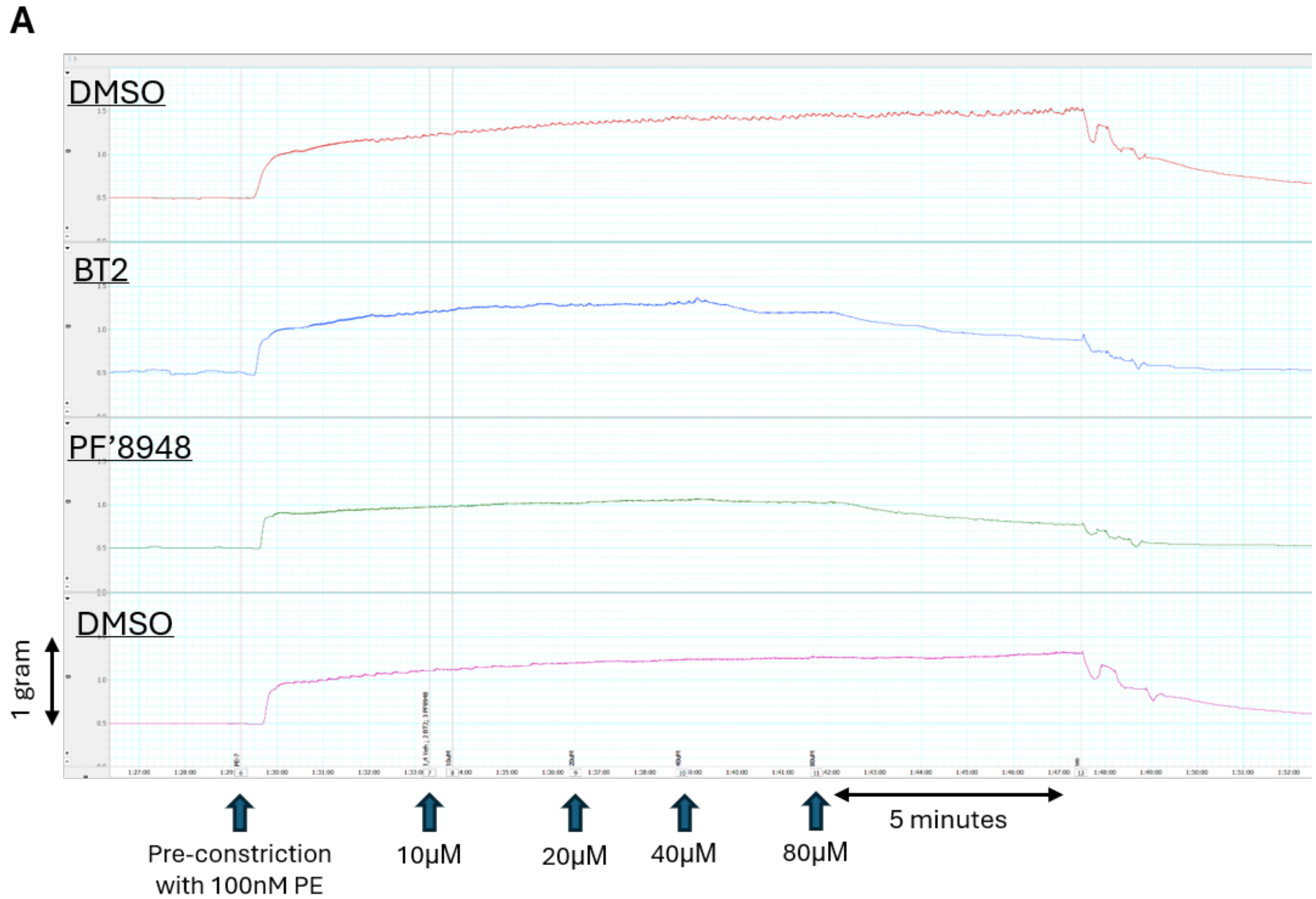

Supplemental Figure 1. Representative myograph records.

bioRxiv preprint doi: https://doi.org/10.64898/2026.10.06.757051; this version posted October 11, 2026. The copyright holder for this preprint (which was not certified by peer review) is the author/funder, who has granted bioRxiv a license to display the preprint in perpetuity. It is made available under a CC-BY 4.0 International license.

Figure S2

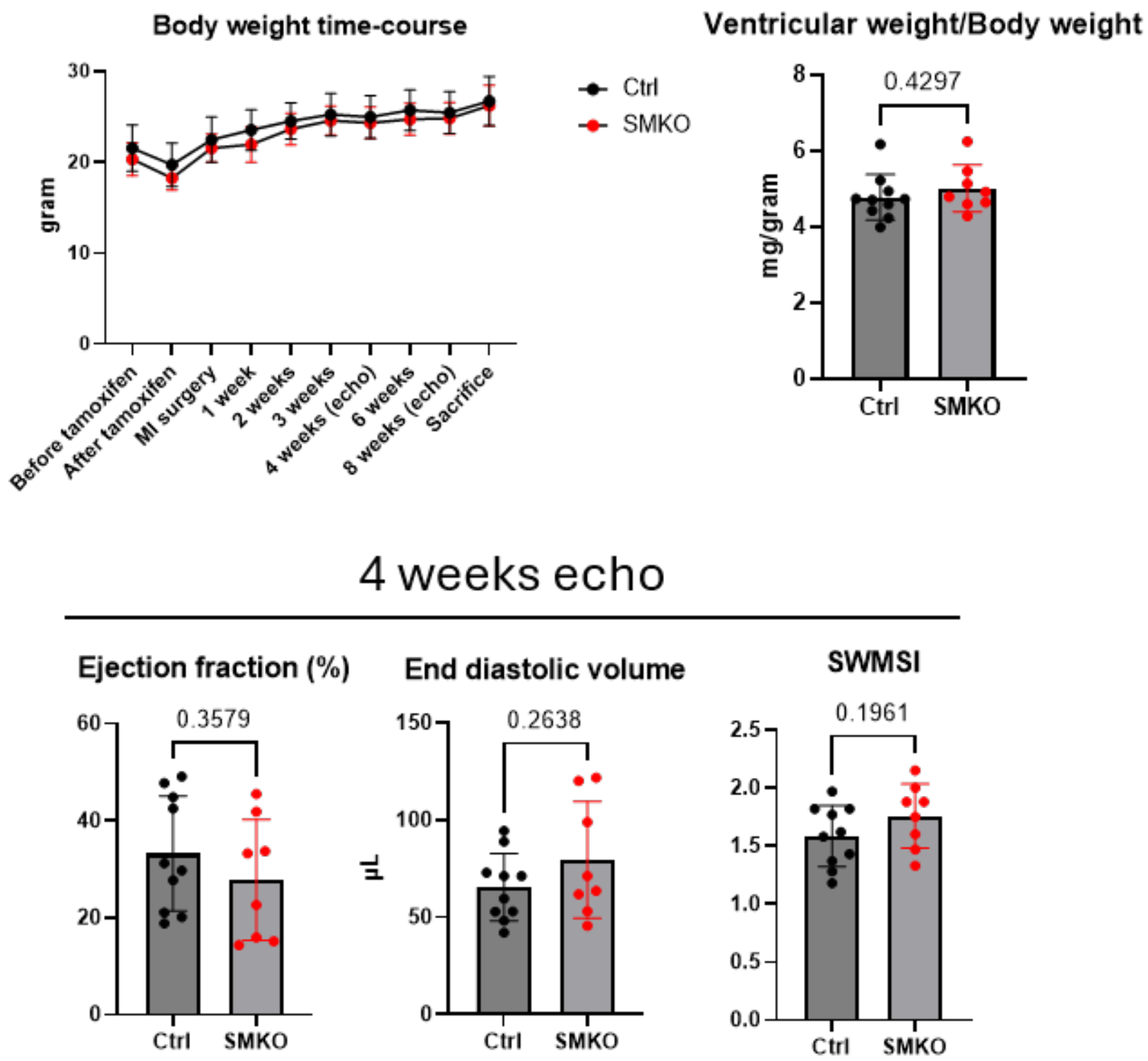

Supplemental Figure 2. Body weight, heart weight, and echo data 4 weeks after myocardial infarction. Unpaired t-test with Welch's correction was performed to compare SMKO and Ctrl.

bioRxiv preprint doi: https://doi.org/10.64898/2026.10.06.757051; this version posted October 11, 2026. The copyright holder for this preprint (which was not certified by peer review) is the author/funder, who has granted bioRxiv a license to display the preprint in perpetuity. It is made available under a CC-BY 4.0 International license.

Figure S3

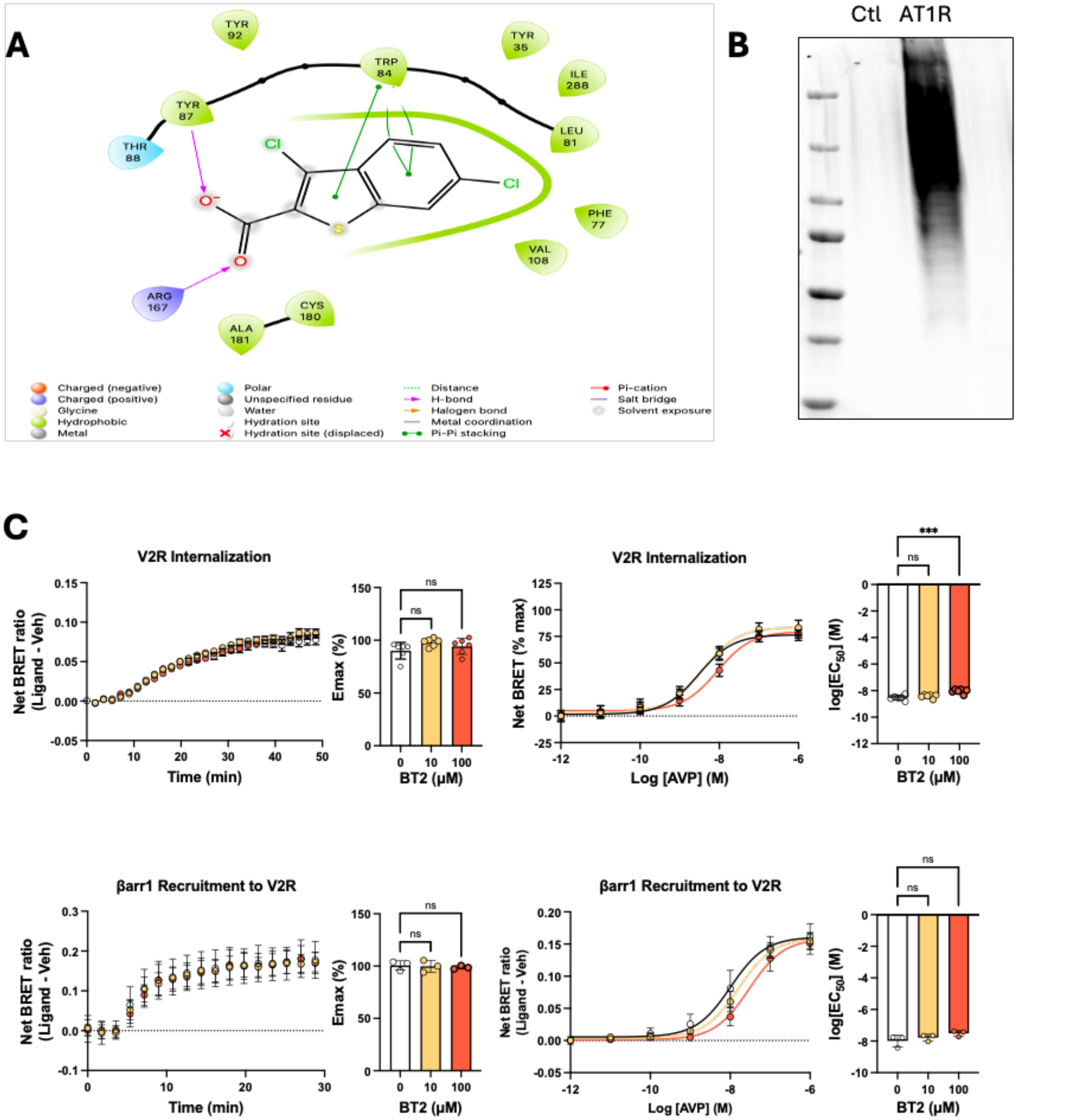

Supplemental Figure 3. A) Predicted docking of BT2 in the orthosteric pocket of AT1R in the inactive conformation. **B)** Western blot for AT1R of control (Ctl) Expi293 membranes and those overexpressing AT1R (AT1R). **C**) HEK293T cells expressing V2R were pretreated with BT2 or vehicle before stimulation with arginine vasopressin (AVP). V2R internalization was assessed by BRET between V2R-RlucII and the endosomal marker 2×FYVE-mVenus (n = 6 independent experiments), and β-arrestin 1 recruitment was assessed by BRET between V2R-RlucII and β-arrestin 1-YFP (n = 3 independent experiments). Data are mean ± SEM. Statistical comparisons
